# Investigating a strategy for quantifying schistosome infection levels in preschool-aged children using prevalence data from school-aged children

**DOI:** 10.1371/journal.pntd.0008650

**Published:** 2020-10-01

**Authors:** Rivka M. Lim, Mark E. J. Woolhouse, Takafira Mduluza, Margo Chase-Topping, Derick N. M. Osakunor, Lester Chitsulo, Francisca Mutapi

**Affiliations:** 1 Institute of Immunology & Infection Research, University of Edinburgh, Ashworth Laboratories, Edinburgh, United Kingdom; 2 Usher Institute, University of Edinburgh, Ashworth Laboratories, Edinburgh, United Kingdom; 3 NIHR Global Health Research Unit Tackling Infections to Benefit Africa (TIBA) at the University of Edinburgh, Ashworth Laboratories, Edinburgh, United Kingdom; 4 Department of Biochemistry, University of Zimbabwe, Mount Pleasant, Harare, Zimbabwe; 5 Roslin Institute, Easter Bush, Midlothian, United Kingdom; 6 Lilongwe, Malawi; Federal University of Agriculture Abeokuta, NIGERIA

## Abstract

In 2012, the World Health Organisation (WHO) set out a roadmap for eliminating schistosomiasis as a public health problem by 2025. To achieve this target, preschool-aged children (PSAC; aged 6 years and below) will need to be included in schistosomiasis treatment programmes. As the global community discusses the tools and approaches for treating this group, one of the main questions that remains unanswered is how to quantify infection in this age group to inform treatment strategies. The aim of this study was thus to determine whether a relationship exists between levels of schistosome infection in PSAC and school-aged children (SAC), that can be used to determine unknown schistosome infection prevalence levels in PSAC. A systematic search of publications reporting schistosomiasis prevalence in African PSAC and SAC was conducted. The search strategy was formulated using the PRISMA guidelines and SPIDER search strategy tool. The published data was subjected to regression analysis to determine if a relationship exists between infection levels in PSAC and SAC. The interaction between SAC and community treatment history was also entered in the regression model to determine if treatment history significantly affected the relationship between PSAC and SAC prevalence. The results showed that a significant positive relationship exists between infection prevalence levels in PSAC and SAC for *Schistosoma mansoni (r* = 0.812, *df* (88, 1), *p* = <0.0001) *and S*. *haematobium (r* = 0.786, *df* (53, 1), *p* = <0.0001). The relationship was still significant after allowing for diagnostic method, treatment history, and the African sub-region where the study was conducted (*S*. *mansoni*: *F* = 25.63, *df* (88, 9), *p* = <0.0001; *S*. *haematobium*: *F* = 10.20, *df* (53, 10), *p* = <0.0001). Using the regression equation for PSAC and SAC prevalence, over 90% of the PSAC prevalence studies were placed in the correct WHO classifications category based on the SAC levels, regardless of treatment history. The study indicated that schistosome prevalence in SAC can be extended as a proxy for infection levels in PSAC, extending on its current use in the adult population. SAC prevalence data could identify where there is a need to accelerate and facilitate the treatment of PSAC for schistosomiasis in Africa.

## Author summary

Preschool-aged children (PSAC), i.e. aged ≤ 6 years, are not included in preventative chemotherapy programmes, currently advocated by the WHO for the treatment of schistosomiasis. This is due to the lack of a paediatric formulation of the drug of choice, praziquantel, and the current guidelines which requires diagnosis before treatment. As the global community prepares for the deployment of a new paediatric formulation of praziquantel, there is a need to find a strategy to quantify infection in this age group. In schistosome endemic areas, infection levels in school-aged children (SAC) are already used to inform infection levels in the community. Thus, we investigated the relationship between SAC and PSAC schistosome prevalence levels within the same community, to determine if data from SAC could be used to predict infection levels in PSAC. Our results show that PSAC prevalence levels are significantly correlated with SAC prevalence levels. Our findings are applicable to communities that have received preventative chemotherapy in SAC or in SAC and adults, as well as those that have not received any. Our study indicates that it is possible to extrapolate PSAC prevalence levels from SAC prevalence, and to make a treatment decision on that basis.

## Introduction

Schistosomiasis is a widespread parasitic disease found in tropical and subtropical areas [1]. The World Health Organization (WHO) estimated that at least 206 million people worldwide required treatment for schistosomiasis in 2016, with at least 91% living in Africa [2]. The most widely used treatment for schistosomiasis is the antihelminthic drug of choice, praziquantel (PZQ), which is both safe and efficacious against adult worms [3]. Currently, schistosomiasis is controlled through preventative chemotherapy, targeting school-aged children (SAC) who are treated with PZQ through mass drug administration (MDA). The frequency of treatment follows guidelines from the WHO, which are based on the schistosome endemicity of the area rather than individual infection status [4]. The endemicity of the area is determined by quantifying schistosome infection prevalence in SAC, following sampling of a group of the children. The reason SAC are used for determining community schistosome endemicity and are the primary target of MDA is that they have been shown to have the highest prevalence of infection and are easily accessible in schools [5, 6].

As of November 2018, 29 of the 41 African countries requiring preventive chemotherapy have implemented MDA preventative chemotherapy in SAC; approximately 75 million children have already received treatment [7]. However, none of these programmes include preschool-aged children (PSAC; aged 6 years and below). In endemic areas, children can be infected as early as 1 year old, and the burden of infection as well as disease morbidity increases with age [8, 9]. Several authors have highlighted the gap in treatment strategies, created by the exclusion of PSAC (reviewed by Stothard *et al*. 2013) [10]. This creates a health inequity for approximately 50 million African PSAC exposed to schistosomiasis [11].

In order to eliminate schistosomiasis as a public health problem by 2025, based on the WHO roadmap on neglected tropical diseases (NTDs) [12], there is a need to include PSAC in treatment programmes. The WHO have responded by recommending the treatment of PSAC [13], and also calling for the development of a paediatric formulation of PZQ, suitable for treating PSAC. There is now a paediatric formulation of PZQ under development (currently undergoing Phase III clinical trials) targeting children aged 3 months to 6 years. Thus, in preparation for inclusion of PSAC in treatment programmes, either as part of MDA or treatment upon diagnosis, there is a need to develop a strategy for quantifying infection in this age group.

Unlike SAC who are readily accessible in schools, PSAC are not often as accessible, and conducting mapping exercises to quantify their infection levels can be challenging and costly. Therefore, we aimed to determine if SAC infection prevalence data could be used to determine infection prevalence in PSAC. In this study, we investigated the relationship between schistosome prevalence levels in SAC and PSAC, to determine if SAC infection levels in Africa can be used as a predictor of the prevalence levels in PSAC from the same community [13, 14].

## Methods

This study focused on published manuscripts reporting schistosome infection prevalence, derived from parasitological detection of schistosome eggs excreted in urine or stool. A systematic search of publications that reported schistosomiasis prevalence in PSAC and SAC in Africa was carried out. The search strategy was formulated using the PRISMA guideline (Preferred Reporting Items for Systematic Reviews and Meta-Analysis) [15], and the SPIDER search strategy tool (Sample, Phenomenon of Interest, Design, Evaluation, Research type) [16] in order to formulate a search question (see Table 1).

**Table 1:** Search terms created using the SPIDER search strategy.

**Sample**	Children (preschool and school age)
**Phenomenon of interest**	Schistosomiaisis infection, either *S*. *haematobium* or *S*. *mansoni*
**Design**	Cross-section, survey
**Evaluation**	Egg count in urine or stool
**Research type**	Not included in search criteria but reviews and meta-analysis were removed during screening
**Terms used in search**	
**S**	Children OR Preschool OR Pre - school OR Infant OR Infants OR PSAC OR SAC
	AND
**PI**	Schisto* OR Bilharzia
	AND
**D**	Cross section OR Cross-section OR Cross-sectional OR Survey OR Prevalence
	AND
**E**	Urine OR Stool OR Katz OR egg OR eggs
	
**R**	N/A

Published studies were identified using three online databases: PubMed (conception to 30^th^ October 2018), Embase (1980–2018, week 44) and Web of Science (1983 – 30^th^ October 2018). The results were filtered to show only those published in English.

### Criteria for inclusion and exclusion of studies

Results from the literature searches were consolidated and extracted using EndNote referencing software (X8.01). Endnote and a Python script were used to match and remove duplicate titles.

Eligibility for inclusion was based on the following criteria: i) the survey was carried out in Africa, ii) it was published/written in English, iii) it contained prevalence data for PSAC, SAC or both, iv) the sample size was clearly stated, v) the sample size for each age group was above 10 people, vi) parasites identified were either *S*. *mansoni* or *S*. *haematobium*, vii) diagnosis was via egg count in stool or urine samples, viii) the age ranges were clearly defined, and ix) PSAC age range was between 0 up to 6 years and SAC age range was between 6–16 years.

The studies were further assessed using the following criteria: recruitment of participants was random and non-biased, and sample collection and diagnostics was clearly explained and carried out according to standard protocols [6]. Any studies not meeting these criteria were excluded from the analyses. Data were entered based on prevalence of schistosomiasis per water source/village, therefore one citation could yield multiple datasets.

### Quality assessment

The quality of each study included in the analysis was assessed using a graded scale. Each study was scored either 1 (yes) or 0 (no) against multiple criteria. There were 7 criteria–i) population sampling methods described; ii) results stratified by gender; iii) a description of laboratory diagnostics included; iv) multiple samples taken on different days; v) multiple tests of a single sample carried out (*S*. *mansoni* only); vi) water source and/or the sanitary conditions described; and vii) statistical analysis described in the methods. The scores were added together and the overall score per article graded as follows: high 6–7, medium 3–5, and low 0–2 for *S*. *mansoni*, and for *S*. *haematobium*, high 5–6, medium 3–4 and low 0–2. If any of the studies scored low on quality assessment, the analysis was conducted with and without the study, to determine if it had an effect on the overall results.

### Study variables

Study variables extracted from the publications were: sample size, age range, egg detection method used for schistosome diagnosis, schistosome treatment history of the study communities, and the antihelminthic drug used for the treatment. The studies used different age ranges for defining PSAC and SAC. For this study PSAC was defined as ages 0–6 years and SAC were defined as 6 years up to 16 years of age. Techniques for detecting eggs in stool for *S*. *mansoni* diagnosis were i) formol-ether sedimentation [17], ii) the Bell Method [18], and iii) Kato-Katz thick smear [19]. For *S*. *haematobium*, egg techniques reported were urine sedimentation and filtration methods [20]. Studies were included in the analysis providing the paired PSAC and SAC infection were diagnosed using the same technique. History of schistosome treatment of the community or the study participants was recorded and included in the statistical analyses; this information was available at a community/group level but not individual participant level. Four categories of treatment history were recorded; 1) No treatment taken place prior to study (i.e. no recorded mass treatment in the past 50 years), 2) Treatment currently underway, 3) Treatment has been recorded within the past 5 years, and 4) No information available about treatment history. If the study did not state whether the community had undergone previous MDA, the WHO Preventative Chemotherapy Treatment (PCT) databank was used to assess whether treatment strategies were in place in this country or had taken place in the past 5 years [21].

### Data extraction

All eligible articles were read in full. The following information was extracted and tabulated (Microsoft Excel); first author name, year of publication, dates of survey, country, African sub-region, sample size, age range, male to female ratio, overall prevalence of infection, infection intensity, male and female infection prevalence, standard deviation, standard error of the mean and 95% confidence intervals, previous schistosome treatment history and method used for collecting the eggs for schistosomiasis diagnosis.

If the data were presented in smaller age ranges, for example, one of the studies specified groups were 6–10 years and 10–16 years, the sample sizes were added together, and from this combined group an average prevalence was calculated. When the age groups exceeded the limits defined by this study e.g. above 6 years old for PSAC or above 16 years old for SAC, the study was removed from the analysis. Furthermore, if the SAC group was partitioned into two, and the older group exceeded the upper limit of the grouping, only the younger group within the SAC was included. Where the published data were presented in graphs, Data-Thief software (Data Thief III), a highly accurate data extraction tool, was used to extract the exact numerical data [22].

Two categories of data were identified, Category 1 in which both PSAC and SAC data come from the same study, and Category 2 in which the data were from two separate studies. Category 2 studies were matched using the following additional criteria: the surveys were carried out within one year of each other, the population used the same water source, and the parasitology diagnostic techniques were the same.

### Statistical analysis

Data analysis was carried out using MINITAB (version 18). As the infection data for both SAC and PSAC were provided as prevalences (recorded as percentage data), both variables were transformed using the arcsine square root transformation to satisfy the assumptions for parametric tests, and to avoid resultant negative prevalence values post-transformation [23]. We determined whether or not data satisfied the parametric assumptions through analysing the residuals (e.g. normality plots and histogram). We also validated the regression model by analysing the goodness of fit of the regression and analysing whether the regression residuals are random.

To determine the relationship between PSAC and SAC, Pearson’s correlation and linear regression without any confounding factors were used. Thereafter, the relationship was analysed through linear regression, allowing for the effect of confounding categorical variables: the African sub-region where the study was conducted (North, South, East, West and Central (see https://unstats.un.org/unsd/methodology/m49/), diagnostic technique (*S*. *mansoni*–Kato katz, Formol-ether or Bell, and *S*. *haematobium*–Filtration or sedimentation), and history of schistosomiasis treatment (No previous treatment in population, treatment within the past 5 years, treatment currently underway and history not given). Also included in the model was the interaction between SAC infection prevalence and treatment history, to determine if the relationship between the two prevalences differed according to the treatment history of the study population.

The relationship between PSAC and SAC was tested for (after allowing for the effects of the above mentioned confounding variables), using adjusted sums of squares to calculate the *F*-value. Adherence to the assumptions of parametric tests was confirmed by a series of residuals plots including histogram plots, residual vs. fits and residuals vs. plots. The significance value was p <0.05.

The goodness of fit of the model to the data was recorded as the *r*-squared value, and the regression coefficient’s 95% confidence intervals were calculated using the equation:

95% CI = *b* ± *tc*_(n-2)_ x SE

Where;

*b* = regression coefficient

*tc* = Critical T = T value at 0.05 for n-2 degrees of freedom

n = study sample size.

SE = standard error of the coefficient

The 95% prediction band of the regression line were generated and plotted in GraphPad (Version 8). Statistical analyses were conducted with and without the data from the studies scoring low on the quality assessment (see S3 Table and S4 Table). This did not affect the result, and so the studies were included in the analysis.

## Results

### Literature search

**Fig 1:**
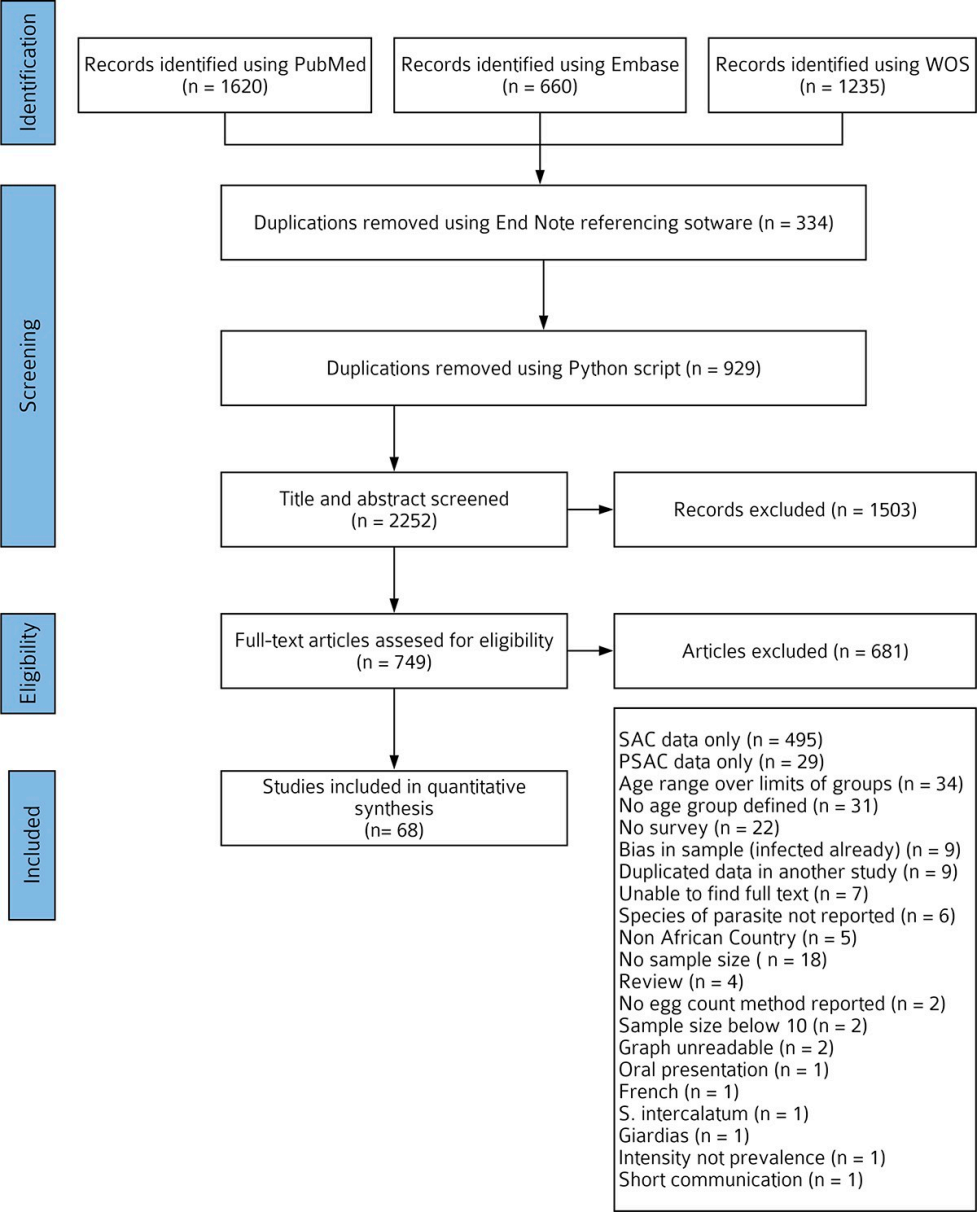
Flowchart for search and selection of included studies.

The selection process is presented in Fig 1. Literature searches of online databases returned 3515 studies. 1263 duplications were identified and removed, leaving 2252 unique studies. After the screening and full text read, 68 studies and 143 datasets (89 for *S*. *mansoni* and 54 for *S*. *haematobium*) were used in the analysis.

### Eligible studies

**Fig 2:**
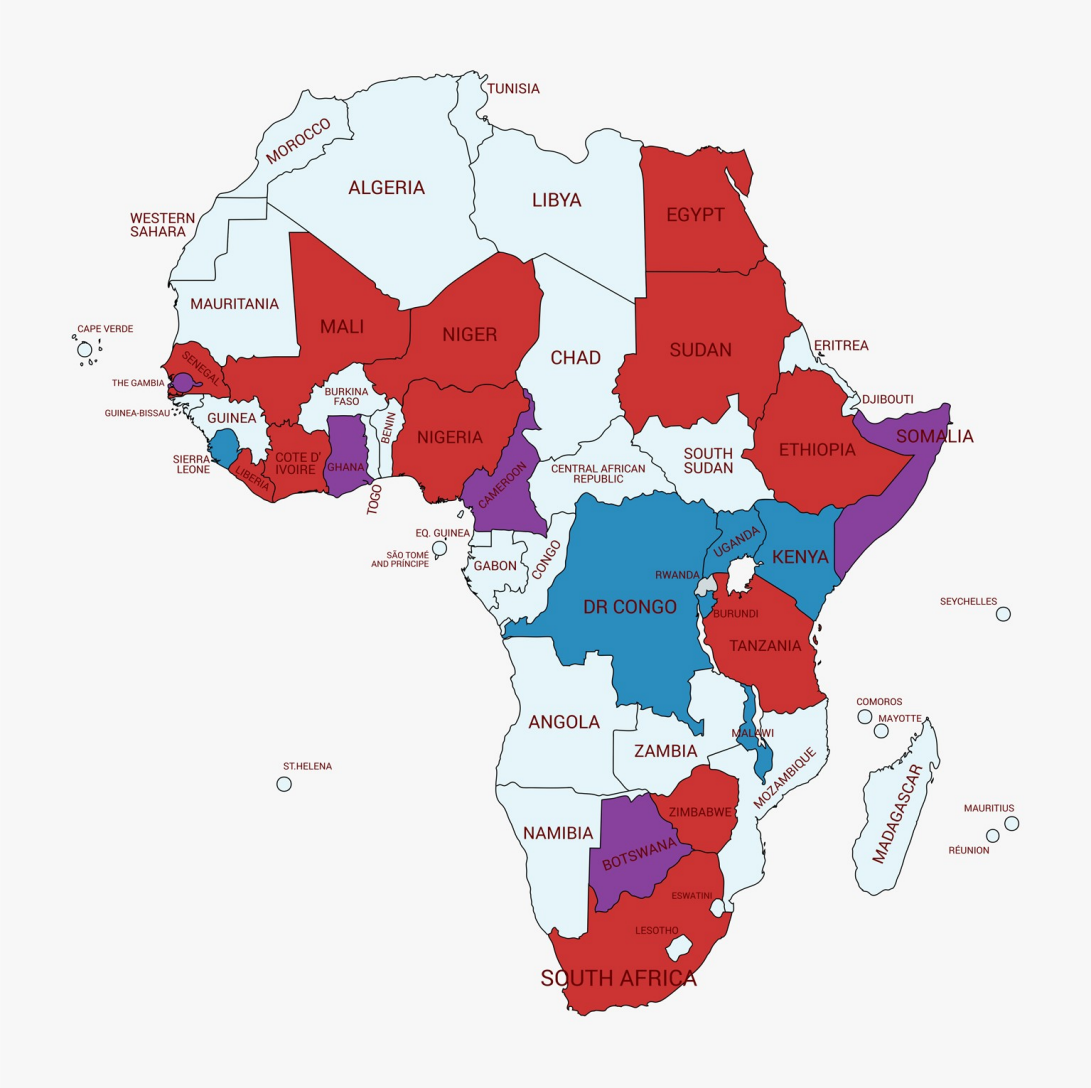
Map of African countries included in the analysis. The map shows the countries where the data came from, partitioned by schistosome species, red = both *S*. *mansoni* and *S*. *haematobium* data present, blue = *S*. *mansoni* only, purple = *S*. *haematobium* only and pale blue = no data used from these countries. The map was made using the online MAPCHART software package (https://mapchart.net/africa.html).

In total, 23 African countries had prevalence data which could be used in the analysis (Table 2) and these were spread across the continent (Fig 2).

**Table 2:** Publications and data sets included in analysis, broken down by country of origin.

	*S*. *mansoni*		*S*. *haematobium*	
Country	Publications	Data sets	Publications	Data sets
Botswana	0	0	1	1
Burundi	1	1	0	0
Cameroon	0	0	2	5
Cote d'Ivoire	4	7	5	6
Egypt	5	8	2	3
Ethiopia	4	8	1	1
The Gambia	0	0	1	1
Ghana	0	0	3	3	
Kenya	5	9	0	0
Liberia	1	1	1	1
Malawi	1	1	0	0
Mali	1	1	1	1
Niger	1	1	3	3
Nigeria	1	1	7	7
Senegal	2	2	2	2
Sierra Leone	2	7	0	0
Somalia	0	0	1	3
South Africa	1	1	1	1
Sudan	1	5	2	6
Tanzania	1	1	3	2
Uganda	4	5	0	0
Zaire (DRC)	1	29	0	0
Zimbabwe	1	1	4	8

### *Schistosoma mansoni* extracted data

There were 89 paired data points for *S*. *mansoni* extracted from 40 manuscripts with surveys between 1974 and 2018. S1 Table, shows characteristics of all studies used in the meta-analysis for *S*. *mansoni* infection in PSAC and SAC. Sample sizes ranged from 11 to 1122 in PSAC (mean = 145, median = 68). Sample sizes in SAC ranged from 12 to 2856 (mean = 333, median = 107). The prevalence of infection ranged from 0–100% in PSAC (mean = 25%, median = 15%, the interquartile range (IQR) = 33.1, standard deviation (SD) = 25.8) and 2.5–100% in SAC, (mean = 50%, median = 47%, IQR = 58.6, SD = 30.3).

For infection detection, the Kato-Katz technique was used in 86 of the 89 (96.6%) investigations, 2 (2.2%) used the formol-ether technique, and 1 (1.1%) used the Bell Method for egg detection in stool. Out of 89 data points, no treatment had previously taken place in 72 (80.9%) communities, 13 (14.6%) of the communities reported treatment currently underway, 1 (1.1%) study reported one round of MDA 2 years previously but no further treatment had taken place, and 2 communities (2.2%) did not report whether control strategies had taken place or not. For the *S*. *mansoni* analysis, 77 were Category 1 (data was from the same study) and 11 were from Category 2 (data was from two separate studies and adhere to the additional inclusion criteria).

### *Schistosoma haematobium* extracted data

For *S*. *haematobium*, there were 54 paired data points from 39 manuscripts, and surveys occurred between 1962 and 2016. S2 Table shows characteristics of all studies that investigated *S*. *haematobium* prevalence in PSAC and SAC. Sample sizes ranged from 11 to 1018 in PSAC (mean = 141, median = 78). Sample sizes of SAC ranged from 21 and 4326 (mean = 557, median = 215). The prevalence of infection ranged from 0–88% in PSAC (mean = 26.2%, median = 19.9%, IQR = 29.5, SD = 23.84) and 0–99% in SAC (mean = 51.5%, median = 54.2%, IQR = 36.7, SD = 27.9). Two diagnostic methods were included in the analysis, the filtration technique [20] was used in 36 of the 54 investigations, and the sedimentation technique was used in the remaining 18 studies.

No treatment had taken place prior to the study in 41 out of 54 (75.9%) of the data sets analysed, 8 out of 54 (14.8%) reported treatment currently underway, 1 (1.9%) study reported treatment of previously positive SAC with Niridazole, about 5 years previously, and 4 (7.4%) had no information on previous treatment status. For the *S*. *haematobium* analysis, 51 were Category 1, and 3 were from Category 2.

The sample sizes for the different independent variables are given in Table 3. Age range for PSAC across the published studies for both schistosome species was <1 up to 6 years, and for SAC, age range was 6–16 years. While for all studies PSAC sample sizes were lower than SAC sample sizes, the sample sizes recorded in the published studies for both species were correlated in PSAC and SAC i.e. those studies with low sample sizes for PSAC also had low sample sizes for SAC and the converse was true. Therefore, the regression analysis was weighted with the square root of the PSAC sample size.

**Table 3:** Summary of sample sizes for categories.

Variable	Categories	*Schistosoma mansoni*	*Schistosoma haematobium*
African Region	North	13	9
	South	1	2
	East	26	14
	West	20	24
	Central	29	5
Treatment History	No previous treatment	73	41
	Currently undergoing treatment	13	8
	MDA previously conducted in the area	1	1
	Treatment history information not provided	2	4
Diagnostic Method	Urine Filtration	N/A	36
	Sedimentation	N/A	18
	Kato Katz	87	N/A
	Formol Ether Technique	1	N/A
	Bell	1	N/A

N/A = does not apply

### Relationship in schistosome infection prevalence between preschool-aged children (PSAC) and school-aged children (SAC)

The Pearson correlation showed a linear relationship between PSAC infection prevalences and SAC infection prevalence in the same communities for both *S*.*mansoni* (*r* = 0.812, *df* = 1, *p* = <0.0001) and *S*. *haematobium* (*r* = 0.786, *df* = 1, *p* = <0.0001). This was subsequently analysed through linear regression.

**Fig 3:**
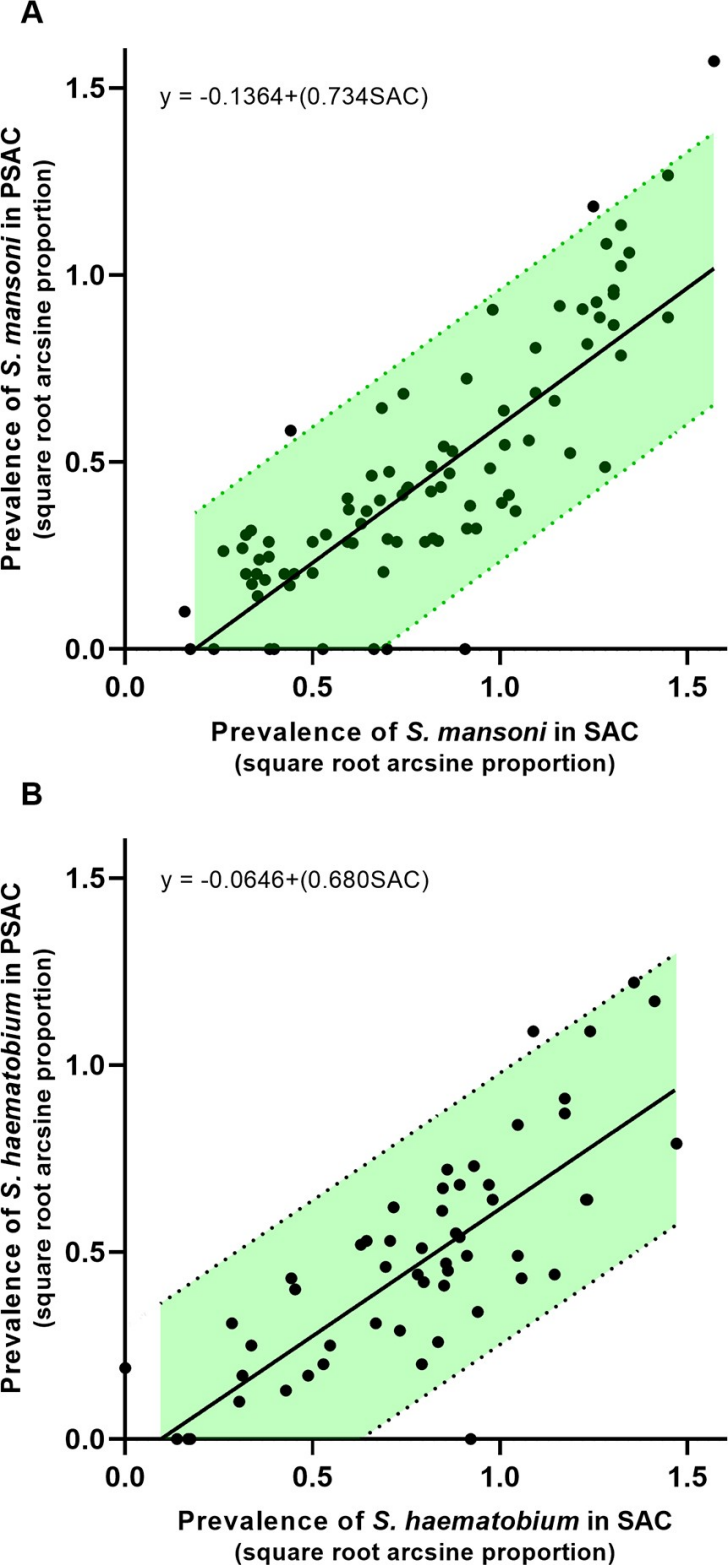
Scatterplots of PSAC vs SAC prevalence with 95% confidence band of the regression line for A) *S*. *mansoni* and B) *S*. *haematobium*. Fitted line is from the linear regression analysis.

The simple regression analysis was carried out without the effect of confounding variables, and was weighted by the square root of PSAC sample size. This showed a significant relationship between *S*. *mansoni* PSAC and SAC prevalences, with 65.9% of the variation in *S*. *mansoni* PSAC prevalence being explained by the model with SAC infection prevalence alone (95% CI = 54.7–77.1). Similarly, the regression analysis showed a significant linear relationship between *S*. *haematobium* PSAC and SAC prevalences with 61.8% of the variation in *S*. *haematobium* PSAC prevalence being explained by the model with SAC infection prevalence alone (95% CI = 46.4–77.2). The simple relationship between PSAC and SAC prevalences is shown in Fig 3A and 3B, and the results of the regression analysis are summarised in Table 4 (*S*. *mansoni*) and Table 5 (*S*. *haematobium*).

**Table 4:** Analysis of variance and coefficients—*S*. *mansoni*, *basic regression weighted by square root of* PSAC*n*.

**Source**			**df**	**F-value**	**P- value**
Regression			88 (1)	168.17	<0.001
Prevalence in SAC			88 (1)	168.17	<0.001
**Coefficients**	**Coef**	**SE Coef**	**95% CI Lower**	**95% CI Upper**	**P- value**
Constant	-0.136	0.049	-0.265	-0.007	0.006
Prevalence in SAC	0.734	0.056	0.623	0.845	<0.001

**Abbreviations**: n–sample size, PSAC–preschool age children, SAC–School age children-square root arcsine transformed, df- degrees of freedom, Coef–coefficient, SE–Standard error.

Mathematical equation for the model: PSAC prevalence = *A* + *b*SAC prevalence

Where *A* is the constant coefficient and *b* is the Prevalence in SAC coefficient

Therefore: PSAC prevalence = -0.136 + 0.734 x SAC prevalence

**Table 5:** Analysis of variance and coefficients—*S*. *haematobium*, *basic regression weighted by square root of* PSAC*n*.

**Source**			**df**	**F-value**	**P- value**
Regression			54 (1)	84.28	<0.001
Prevalence in SAC			54 (1)	84.28	<0.001
**Coefficients**	**Coef**	**SE Coef**	**95% CI Lower**	**95% CI Upper**	**P- value**
Constant	-0.065	0.062	-0.189	0.059	0.300
Prevalence in SAC	0.680	0.074	0.531	0.828	<0.001

**Abbreviations**: n–sample size, PSAC–preschool age children, SAC–School age children-square root arcsine transformed, df- degrees of freedom, Coef–coefficient, SE–Standard error.

Mathematical equation for the model: PSAC prevalence = *A* + *b*SAC prevalence

Where *A* is the constant coefficient and *b* is the Prevalence in SAC coefficient

Therefore: PSAC prevalence = -0.065 + 0.680 x SAC prevalence

**Fig 4:**
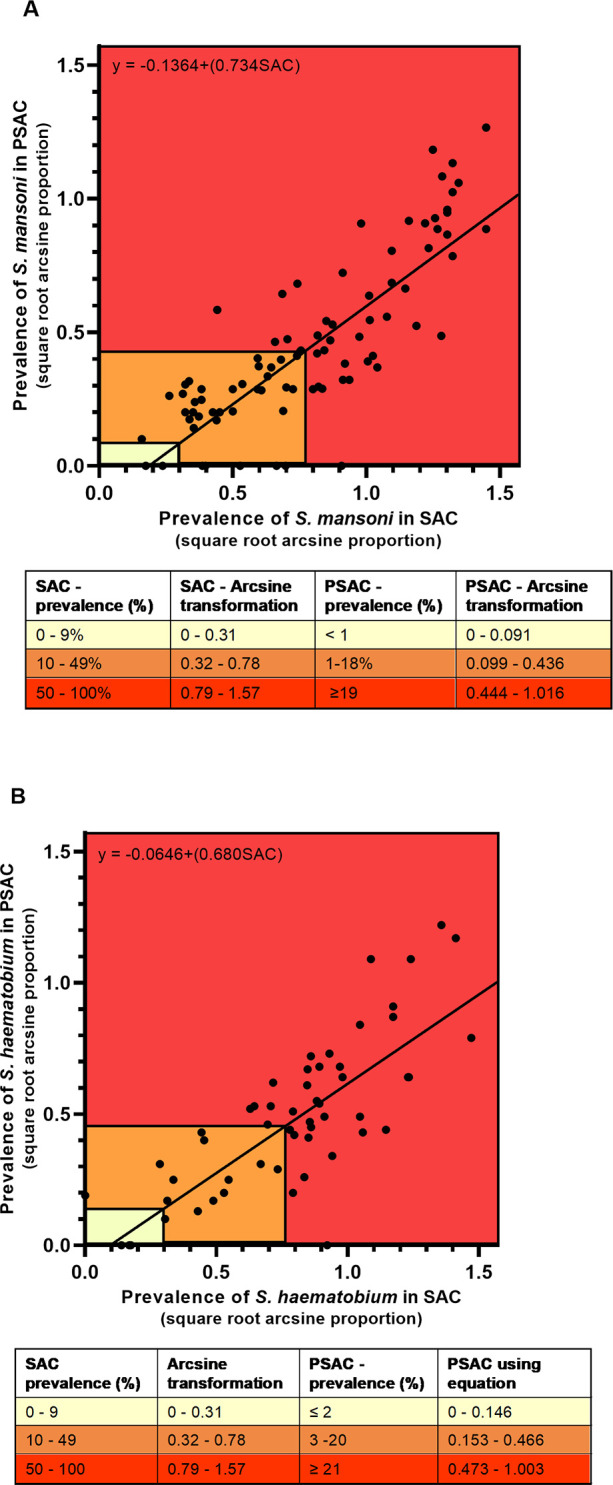
Scatterplots of PSAC vs SAC prevalence demarcated into the WHO prevalence classes for A) *S*. *mansoni* and B) *S*. *haematobium*. The classes are shown according to SAC prevalence levels showing the corresponding PSAC level on the Y-axis. Red = High, Orange = Moderate, Yellow = Low. The fitted line is from the linear regression analysis.

The WHO provides guidelines [4] for the optimal frequency of schistosome treatment of SAC, by assessing the prevalence of infection or visible haematuria in a community. We therefore coded the infection prevalence in PSAC using the guidelines for SAC (low, moderate and high) to indicate levels of infection in PSAC, which would be captured under those categories (see Fig 4). It can be seen that when SAC prevalence levels are low, there is a low infection prevalence in PSAC, whereas when infection levels are high in SAC, levels in PSAC are moderate to high. There are two instances where PSAC infection levels are higher than in SAC, only one published report for each schistosome species, and the fitted line in Fig 4 predicts that a 100% infection prevalence is reached earlier in SAC than PSAC.

The regression predicts PSAC prevalences for the WHO categories based on the SAC prevalences. For example, when SAC levels are low in *S*. *mansoni*, i.e. 0–9%, the PSAC levels are interpolated to be <1% (Fig 4A). Therefore based on the prediction, we determined the proportion of PSAC studies wrongly classified into a lower infection level, based on the SAC prevalences. Being, misclassified into a lower level is important from a public health perspective, as it means infection levels in PSAC will be underestimated.

For *S*. *mansoni*, the PSAC infection was underestimated in 4/89 study populations (4.5%). One study population (out of 4) had a PSAC prevalence that was classified in the low WHO category based on its SAC prevalence when it had a PSAC prevalence greater than 1% and therefore, should have been in the moderate category. This study reported the same prevalence 6.7% for both SAC and PSAC. Additionally, 3/42 (7.1%) study populations were classified as moderate when their PSAC prevalences would have put them in high infection level. Of these 3 populations, 1 was undergoing treatment. For *S*. *haematobium*, in total 4/54 (7.4%) PSAC study populations were underestimated. One of the study populations (out of 4) had a PSAC prevalence that was classified in the low infection WHO category when it should have been in the moderate category. This study had a PSAC prevalence of 9% which was greater than the SAC prevalence of 7.9%. Further, 4/18 (22.2%) study populations were classified as moderate when their PSAC prevalences would have put them in high infection level. Of these 4 populations, 1 was undergoing treatment.

### Effect of treatment history on the relationship between infection prevalence between preschool-aged children (PSAC) and school age children (SAC)

We conducted a regression analysis, allowing for the African sub-region where the study was conducted for both schistosome species, as well as the parasitology diagnostic method for *S*. *haematobium* only as there was not enough data in the diagnostic categories in the *S*.*mansoni* analysis to give a meaningful investigation. This was to determine if the relationship between PSAC and SAC prevalences varied significantly with the treatment history of the study community. The African sub-region was included in the analysis, as earlier descriptive statistics had indicated that there was heterogeneity in infection prevalences reported by studies from the different countries in Africa; this is confirmed by the significant effect of this variable on PSAC prevalence (see Table 6). We conducted the analysis with and without the studies from the Southern region which had the smallest sample sizes. Excluding these studies did not alter the results significantly, therefore we included all studies in the final analyses.

**Table 6:** Analysis of variance and coefficients—*S*. *mansoni*, *regression weighted by square root of PSACn*.

**Source**			**df**	**F-value**	**P- value**
Regression			88 (9)	25.63	<0.001
Prevalence in SAC			88 (1)	163.15	<0.001
African Regions (North, South, East, West and Central)			88 (4)	4.09	0.005
Treatment History			88 (2)	0.10	0.908
Prevalence in SAC * Treatment History			88 (2)	0.02	0.983
**Coefficients**	**Coef**	**SE Coef**	**95% CI Lower**	**95% CI Upper**	**P- value**
Constant	-0.137	0.065	-0.267	-0.008	0.038
Prevalence in SAC	0.7209	0.0564	0.6086	0.8333	<0.001
**African Regions:**					0.005*
North	0.000	0.000	0.000	0.000	
South	-0.198	0.191	-0.578	0.182	0.303
East	-0.037	0.057	-0.151	0.077	0.519
West	0.055	0.063	-0.071	0.181	0.385
Central	0.132	0.064	0.004	0.260	0.044
**Treatment History:**					0.908*
No previous treatment	0.000	0.000	0.000	0.000	
Treatment underway	-0.060	0.153	-0.364	0.244	0.696
Unknown	0.46	2.65	-4.81	5.73	0.861
SAC *Treatment History:					0.983*
No previous treatment	0.000	0.000	0.000	0.000	
Treatment underway	-0.026	0.200	-0.425	0.373	0.897
Unknown	-1.08	7.71	-16.43	14.28	0.889

**Abbreviations**: n–sample size, PSAC–preschool age children, SAC–School age children-square root arcsine transformed, df- degrees of freedom, Coef–coefficient, SE–Standard error.

*Overall P-value from regression model

Mathematical equation for the model: PSAC prevalence = *A* + *b*SAC prevalence + *c*African Region +*d*Treatment History + *e*SAC*Treatment History

Where *A* is the constant coefficient and *b*,*c*,*d* and *e* are the variable coefficients.

Including the other variables and the interacting term in the model increased the *r*-squared value from 65.9% (95% CI = 54.7–77.1) to 74.5% (95% CI = 65.6–83.4) for *S*. *mansoni* and from 61.8% (95% CI = 46.4–77.2) to 70.3% (95% CI = 57.6–83.0) for *S*. *haematobium*. The correlation between PSAC and SAC prevalence was still significant. The results of this full model analysis are summarised in Tables 6 and 7. This analysis showed that the relationship between PSAC and SAC prevalences was not significantly altered by treatment history (*S*. *mansoni*, *F* value = 0.02, *df* = 88, 2, *p* = 0.983, and *S haematobium*, *F* value = 0.36, *df* = 53, 2, *p* = 0.966).

**Table 7:** Analysis of variance and coefficients—*S*. *haematobium*, *regression weighted by square root of PSACn*.

**Source**			**df**	**F-value**	**P- value**
Regression			53(11)	10.20	<0.0001
Prevalence in SAC			53(1)	57.42	<0.0001
African Regions (North, South, East, West and Central)			53(4)	1.21	0.319
Treatment History			53(2)	0.33	0.721
Diagnostic Technique			53(1)	0.01	0.910
Prevalence in SAC * Treatment History			53(2)	0.36	0.966
**Coefficients**	**Coef**	**SE Coef**	**95% CI Lower**	**95% CI Upper**	**P- value**
Constant	-0.095	0.102	-0.300	0.111	0.359
Prevalence in SAC	0.6776	0.894	0.497	0.8580	<0.0001
**African Regions:**					0.319*
North	0.00	0.00	0.00	0.00	
South	0.007	0.177	-0.350	0.364	0.968
East	0.1080	0.098	-0.090	0.306	0.276
West	0.019	0.102	-0.187	0.224	0.855
Central	0.181	0.181	-0.093	0.454	0.189
**Treatment history:**					0.721*
No previous treatment	0.00	0.00	0.00	0.00	
Treatment underway	-0.074	0.183	-0.433	0.295	0.688
Unknown	-0.483	0.706	-1.908	0.941	0.497
**Diagnostic technique:**					0.910*
Filtration	0.00	0.00	0.00	0.00	
Sedimentation	0.007	0.056	-0.106	0.119	0.614
SAC * Treatment history:					0.966*
No previous treatment	0.00	0.00	0.00	0.00	
Treatment underway	-0.107	0.210	-0.529	0.319	0.614
Unknown	0.544	0.789	-1.047	2.135	0.494

**Abbreviations**: n–sample size, PSAC–preschool age children, SAC–School age children-square root arcsine transformed, df- degrees of freedom, Coef–coefficient, SE–Standard error.

*Overall P-value from regression model

Mathematical equation for the model: PSAC prevalence = *A* + *b*SAC prevalence + *c*African Region +*d*Treatment History + *e*Diagnostic Technique + *f*SAC*Treatment history

Where *A* is the constant coefficient and *b*,*c*,*d*,*e* and *f* are the variable coefficients.

**Fig 5:**
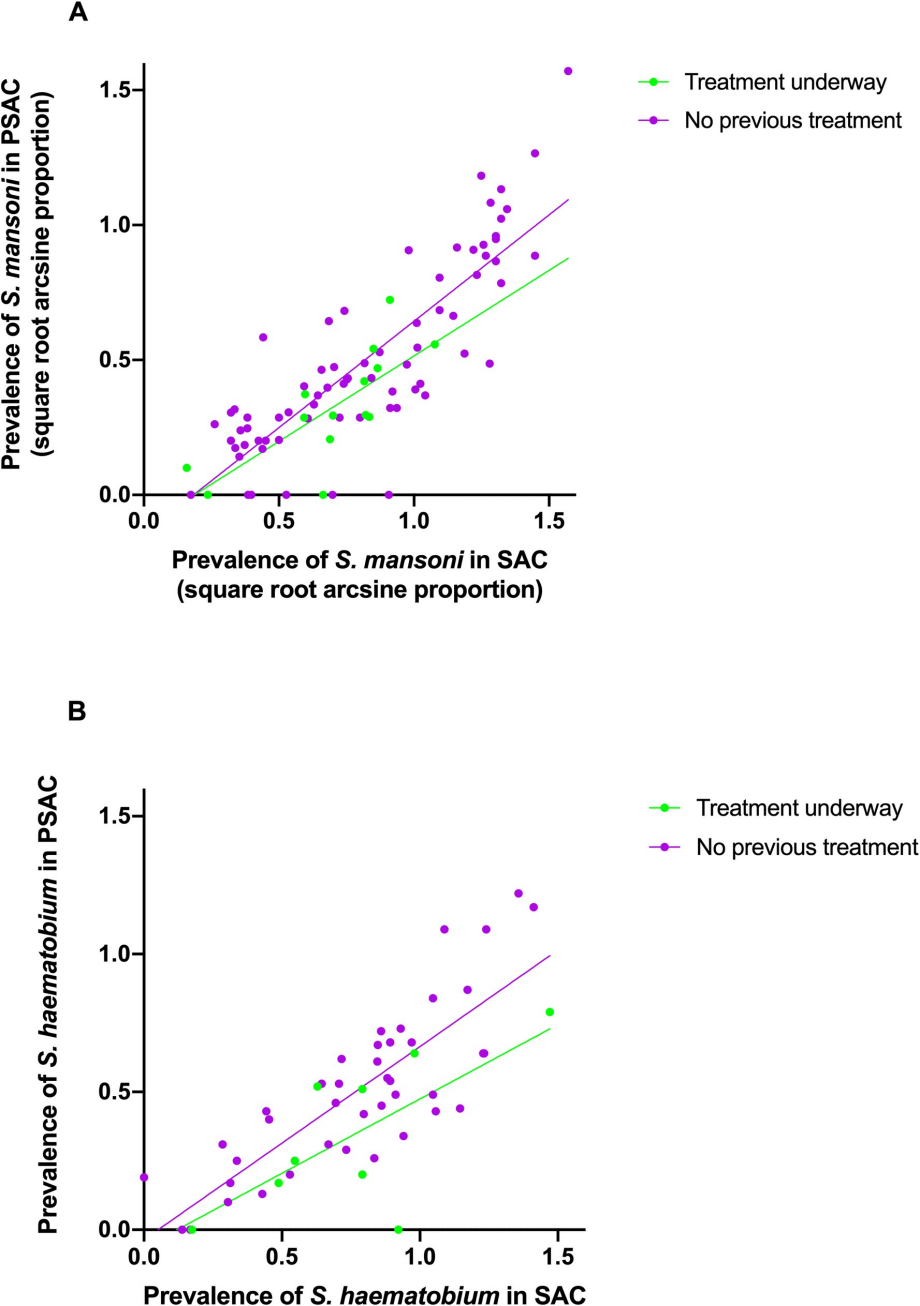
Scatterplots of PSAC vs SAC prevalence partitioned by, treatment history of the SAC for A) *S*. *mansoni* and B) *S*. *haematobium*.

It is worth noting that although statistical significance was not detected for the effect of treatment history on PSAC infection prevalence or for the interaction factor between SAC infection prevalence and treatment history, for both *S*. *mansoni* and *S*. *haematobium*, SAC infection levels in populations where schistosome treatment was underway (e.g. through MDA), consistently mapped to lower PSAC infection levels (see Fig 5).

## Discussion

This is the first study investigating an association between the prevalence of schistosomiasis in African school-aged children (SAC) and preschool-aged children (PSAC). This study is driven by the current need to define strategies to include PSAC in schistosomiasis treatment programmes. The current WHO guidelines for treating PSAC is to treat children on a case-by-case basis upon diagnosis, with the current formulation of PZQ [13]. As the global community prepares for the deployment of the paediatric formulation of PZQ (currently undergoing Phase III clinical trials), and for eventual elimination of schistosomiasis, one of the main operational questions that remain unanswered is how to quantify infection in PSAC, and to identify areas/populations to target for treatment. PSAC are out of the school system and therefore not as easily accessible as SAC. A further operational question is, at what level of schistosome endemicity would it be practical to conduct an infection survey in this age group to diagnose and treat infections? We investigated the relationship between currently available data on SAC and PSAC within the same community to determine if data from SAC, who are the current target of control programmes could be used to predict infection levels in PSAC.

Previous studies have investigated using SAC as a proxy group for community-wide prevalence levels, showing that SAC prevalence is closely related to adult prevalence, and therefore this age group could be used as a proxy group [24, 25]. Our findings confirm that this approach can be extended to quantify infection levels in PSAC. Our results show that PSAC infection levels are significantly correlated with SAC prevalence levels, and indicate that it is possible to extrapolate PSAC prevalence levels from known SAC prevalence and treat them accordingly. This finding is not surprising as SAC tend to use the same water sources as the rest of the community, and their contact with infective water is related to activities of older family members [26], so that when a high proportion of SAC are infected, the community risk of infection accumulates rapidly.

Several countries have embarked on preventative chemotherapy through mass drug administration (MDA). Thus, some of the data we analysed came from communities undergoing MDA programmes targeted at SAC, or in a few cases, targeting both SAC and adults, while others had not experienced MDA in the past 50 years; no current MDA programme includes PSAC. Therefore, we included the SAC, or SAC and adult’s treatment history in our analyses. The current study indicated that the relationship between PSAC and SAC prevalences did not change significantly depending on treatment history of the study population. This finding may be due to the small sample sizes in the study because, albeit not significant in areas undergoing treatment, SAC levels translated to lower PSAC levels than in areas which had not experienced any treatment. In other words, to get the same levels of PSAC prevalence in areas that had undergone treatment, higher SAC prevalence levels were required than when compared to that in areas without treatment, for both *S*. *mansoni* and *S*. *haematobium*. In areas undergoing MDA, treatment of SAC has the potential of lowering levels of contamination of shared water sources with the infection, especially if treatment coverage is high and includes adults in the community; in this case, PSAC are exposed to lower levels of infection. In two examples where treatment was underway, one for S. *mansoni* and one for *S*. *haematobium*, infection prevalence in PSAC was greater than in SAC, presumably because the PSAC are excluded from the MDA programmes.

The maintenance of a relationship between PSAC and SAC prevalences during treatment, is relevant for most countries targeting SAC via preventative chemotherapy, and will need to include PSAC. This will become particularly prominent if the paediatric formulation of PZQ currently undergoing phase III clinical trials becomes available. This means that even data from national treatment impact assessment exercises that are mostly conducted by surveying SAC, are informative for developing strategies for treating PSAC. Thus, PSAC prevalence can be inferred from current levels of SAC prevalence data (as can be found in the Espen WHO database [27]), which can now be used to map prevalence levels of schistosomiasis in PSAC in Africa. Our results suggest that where prevalence of schistosomiasis infection is high in SAC, limited surveys could be used to validate infection in PSAC to justify preventive chemotherapy. This may obviate the need to conduct extensive surveys for schistosomiasis in PSAC. This is also in keeping with the WHO recommendation that given the financial and human resources required for surveys, existing epidemiological data including historical records should be used to generate baseline information for implementing a control strategy [6].

Unsurprisingly the relationship between SAC and PSAC indicates that infection levels in PSAC are zero or undetectable below a certain prevalence of SAC infection. This is due to both the low sensitivity of the widely used parasitological schistosomiasis diagnostic tests in detecting low intensity infections [28], as well as the infection transmission dynamics. Infection accumulates with age, with most children being exposed to infection within their first year of life, mainly as a result of increasing water contact and exposure patterns [29, 30]. While we can determine exposure to infection in the very young children serologically, infection detection by egg counts is less reliable because of the low levels of infection the children carry [29]. There is also the operational challenge of collecting stool and urine samples from the younger PSAC, who may not be toilet-trained; in this study PSAC age ranged from <1 year up to 6 years. Similarly, the number of samples collected would also affect the accuracy of diagnosis and not all studies indicated the number of urine or stool samples collected for the parasitology diagnosis.

Where we had sufficient data to investigate the influence of parasite egg collection methods, we found that for example with *S*. *haematobium*, the choice of egg collection methods, did not affect the PSAC infection prevalence. This is not surprising as both methods relied on enumerating parasite eggs excreted in urine, and thus suffer the same limitations of low sensitivity in people with low infection intensities [30].

Looking at methodological aspects, as with most meta-analyses and literature reviews, some publication bias can occur. Thus, we conducted a quality assessment exercise of the data published in the source reference. We then conducted the analyses both including and excluding the data from studies scoring low on the quality assessment, and as this did not alter the results, the data from all studies satisfying the inclusion criteria were kept in the analysis.

There were other possible analyses that would have been more informative, but sample sizes were too small for the data to be partitioned or analysed by those categories. For example, for both *S*. *mansoni* and *S*. *haematobium*, only one study reported previous, but no current MDA in the SAC. These both had their most recent MDA 2 years prior to the study quantifying SAC and PSAC infection, where the one examining *S*. *haematobium* used Niridazole instead of PZQ. The impact of MDA on the relationship between PSAC and SAC infection levels after cessation of MDA in SAC thus remains unexplored, but may likely not vary significantly due to infection rebound [31]. It would also have been informative to have the data for both SAC and PSAC in each age group, but this more granular data were not presented in the source publication. We attempted to capture some of the heterogeneity in the data by including factors such as the African sub-region where the data were collected, but other factors which could not be determined from the information presented in the publications might also account for the variation in the PSAC infection prevalence (the model explained 74.5% of the variation in *S*. *mansoni* and 70.3% in *S*. *haematobium*) This includes factors such as number of parasitology samples collected, number of slides prepared for diagnosis and age-related water contact. Nonetheless, the strong correlation between the two variables (i.e. PSAC and SAC prevalence) in these heterogeneous studies suggest that when quantifying infection in the same community with less heterogeneity, the SAC infection prevalence should be a more informative proxy for PSAC infection prevalence.

We determined the proportion of study populations classified into a lower infection level i.e. misclassified into a lower WHO classifications category based on the SAC levels. For *S*. *mansoni*, 4/89 (4.5%) of PSAC prevalences were underestimated and therefore misclassified, based on the interpolation of the regression equation. For *S*. *haematobium*, 4/54 (7.4%) of PSAC prevalences were misclassified. There are several reasons that could explain this, an example is treatment history. One of the *S*. *mansoni* studies reported that MDA was underway in SAC, and had similar prevalences in SAC and PSAC. The published source studies reported treatments via MDA but there was no indication if there had been any individual treatment provisions through health facilities. For *S*. *haematobium*, one study reported higher infection prevalence in PSAC than SAC, and the population had not had MDA targeted at the SAC. However, no explanation was given for this observation. The fact that even with the heterogeneities highlighted, over 90% of the studies were classified into the correct WHO classifications category based on the SAC levels from the regression model indicates that the SAC prevalences can be extended as a proxy for infection levels in PSAC as they are already being used for the adult population.

In conclusion, it has already been demonstrated that in schistosome endemic areas, PSAC are at high risk of schistosome infection and disease and therefore should be included in treatment strategies. Here, we have demonstrated that schistosomiasis infection levels in PSAC are highly correlated to levels in SAC, giving the potential for using already existing data from SAC as a predictor of infection levels in PSAC, for both *S*. *mansoni* and *S*. *haematobium*. Critically, we have shown that the relationship between PSAC and SAC infection levels is informative during ongoing community or SAC-targeted treatment, as well as in communities that have not received schistosome treatment. With the increasing concern of the health impact of schistosomiasis in this PSAC, using already existing SAC data to inform PSAC chemotherapy could accelerate and facilitate the treatment of PSAC who are in urgent need of treatment. This will allow endemic countries to move closer towards the goal of eliminating schistosomiasis as a public health burden by 2025 [32].

## Supporting information

S1 Checklist PRISMA checklist. (DOCX)

S1 Table Participant and methodology characteristics of studies included in meta-analysis containing *S*. *mansoni* data. (DOCX)

S2 Table Participant and methodology characteristics of studies included in meta-analysis containing *S*. *haematobium* data. (DOCX)

S3 Table Quality appraisal of articles containing prevalence data of *S*. *mansoni* infection in preschool and school age children in Africa. (DOCX)

S4 Table Quality appraisal of articles containing prevalence data of *Schistosoma haematobium* infection in preschool and school age children in Africa. (DOCX)
