# Supplementary material for: Investigating a strategy for quantifying schistosome infection levels in preschool-aged children using prevalence data from school-aged children
Source: PLoS Negl Trop Dis. 2020 Oct 1;14(10):e0008650. doi: 10.1371/journal.pntd.0008650 (PMC7529243; doi:10.1371/journal.pntd.0008650)
Supplement: S4 Table — (DOCX) [file pntd.0008650.s005.docx]

| **S4 Table.** Quality appraisal of articles containing prevalence data of *Schistosoma haematobium* infection in preschool and school age children in Africa. | | | | | | | | | | | | | | | | | | |
| --- | --- | --- | --- | --- | --- | --- | --- | --- | --- | --- | --- | --- | --- | --- | --- | --- | --- | --- |
| **First Author** | | **Publication Date** | | **Population sampling methods described** | | **Stratified by gender** | | **Description of laboratory diagnostic methods** | | **Multiple urine samples taken on different days?** | | **Water source and/or sanitary conditions described?** | | **Statistical analysis described in methods** | | **Result (out of 6)** | | **Score** |
| Akogun, B. [2] | | 1996 | | 1 | | 1 | | 1 | | 1 | | 1 | | 1 | | 6 | | High |
| Amuta, E.U. [41] | | 2014 | | 1 | | 1 | | 1 | | 0 | | 0 | | 1 | | 4 | | Medium |
| Anosike, J.C. [43] | | 2003 | | 1 | | 1 | | 1 | | 0 | | 1 | | 0 | | 4 | | Medium |
| Anosike. J.C. [42] | | 2006 | | 1 | | 1 | | 1 | | 1 | | 1 | | 1 | | 6 | | High |
| Babiker, S.M. [4] | | 1985 | | 0 | | 1 | | 0 | | 0 | | 0 | | 0 | | 1 | | Low |
| Ben S.A. [44] | | 2017 | | 1 | | 0 | | 1 | | 0 | | 1 | | 1 | | 4 | | Medium |
| Campbell, S.J. [45] | | 2017 | | 1 | | 0 | | 1 | | 0 | | 0 | | 1 | | 3 | | Medium |
| Clark, T. E. [46] | | 1997 | | 0 | | 1 | | 1 | | 0 | | 1 | | 1 | | 4 | | Medium |
| Coulibaly, J.T.[10] | | Dec 2012 | | 1 | | 1 | | 1 | | 1 | | 1 | | 1 | | 6 | | High |
| Coulibaly, J.T. [11] | | July 2012 | | 1 | | 0 | | 1 | | 1 | | 1 | | 1 | | 5 | | High |
| Coulibaly, J.T.[9] | | 2013 | | 1 | | 1 | | 1 | | 0 | | 1 | | 1 | | 5 | | High |
| Coulibaly, J.T. [8] | | 2018 | | 1 | | 1 | | 1 | | 0 | | 1 | | 1 | | 5 | | High |
| De Clercq, D. [12] | | 1994 | | 0 | | 0 | | 1 | | 0 | | 0 | | 0 | | 1 | | Low |
| Deribe, K. [47] | | 2011 | | 1 | | 1 | | 1 | | 0 | | 1 | | 1 | | 5 | | High |
| Duke, B.O.L [48] | | 1976 | | 1 | | 0 | | 1 | | 0 | | 0 | | 0 | | 2 | | Low |
| Ejezie, G.C H. [49] | | 1991 | | 0 | | 1 | | 1 | | 0 | | 1 | | 0 | | 3 | | Medium |
| Garba, A. [17] | | 2010 | | 1 | | 0 | | 1 | | 1 | | 1 | | 1 | | 5 | | High |
| Garba, A. [18] | | 2013 | | 0 | | 1 | | 1 | | 1 | | 1 | | 1 | | 5 | | High |
| Garba, A. [50] | | 2010 | | 0 | | 1 | | 1 | | 1 | | 1 | | 1 | | 5 | High |  |
| Goatly, K.D. [51] | | 1965 | | 0 | | 0 | | 1 | | 0 | | 1 | | 0 | | 2 | Low |  |
| Houmsou, R.S. [52] | | 2016 | | 1 | | 1 | | 1 | | 0 | | 1 | | 1 | | 5 | High |  |
| Kazura, J.W. [27] | | 1985 | | 1 | | 1 | | 1 | | 0 | | 1 | | 1 | | 5 | High |  |
| King, C.L. [53] | | 1982 | | 1 | | 1 | | 1 | | 0 | | 1 | | 0 | | 4 | Medium |  |
| Klumpp, R.K. [54] | | 1987 | | 0 | | 1 | | 1 | | 0 | | 1 | | 0 | | 3 | Medium |  |
| Koura, M. [55] | | 1981 | | 0 | | 1 | | 1 | | 0 | | 1 | | 1 | | 4 | Medium |  |
| Lyons, G.R.L. [56] | | 1974 | | 1 | | 1 | | 1 | | 0 | | 0 | | 0 | | 3 | Medium |  |
| Mansour, N.S. [57] | | 1981 | | 1 | | 1 | | 1 | | 0 | | 1 | | 1 | | 5 | High |  |
| Mekonnen, A. [58] | | 2013 | | 0 | | 1 | | 1 | | 0 | | 1 | | 1 | | 4 | Medium |  |
| Meurs, L. [30] | | 2012 | | 0 | | 0 | | 1 | | 1 | | 1 | | 1 | | 4 | Medium |  |
| Midzi, N. [59] | | 2009 | | 0 | | 0 | | 0 | | 1 | | 1 | | 1 | | 3 | Medium |  |
| Morenikeji, O. [60] | | 2014 | | 1 | | 1 | | 1 | | 0 | | 1 | | 1 | | 5 | High |  |
| Mott, K. E. [61] | | 1985 | | 0 | | 0 | | 1 | | 0 | | 0 | | 0 | | 1 | Low |  |
| Mutapi, F.M. [63] | | 2011 | | 1 | | 0 | | 1 | | 1 | | 0 | | 1 | | 4 | Medium |  |
| Nausch, N. [31] | | 2014 | | 1 | | 1 | | 1 | | 1 | | 1 | | 1 | | 6 | High |  |
| Rudge, J. W. [66] | | 2008 | | 1 | | 1 | | 0 | | 0 | | 1 | | 1 | | 4 | Medium |  |
| Schutte, CHJ. [64] | | 1977 | | 0 | | 1 | | 1 | | 1 | | 1 | | 1 | | 5 | High |  |
| Sousa - Figueiredo, J.C. [65] | | 2013 | | 0 | | 0 | | 1 | | 0 | | 0 | | 1 | | 2 | Low |  |
| Verle, P. [67] | | 1994 | | 0 | | 0 | | 1 | | 1 | | 1 | | 0 | | 3 | Medium |  |
| Wami, W. [62] | | 2015 | | 1 | | 0 | | 1 | | 1 | | 0 | | 1 | | 4 | Medium |  |
| Wilkins, H. A. [68] | | 1985 | | 0 | | 1 | | 0 | | 0 | | 0 | | 0 | | 1 | Low |  |

When an article adequately met the criteria a score of 1 was given, and 0 if it did not. Each article was given a mark out of 6 and allocated a quality score. High were 5-6, medium 3-4, low 0-2.

**References used in analysis**

1. Abdel-Wahab MF, Strickland GT, El-Sahly A, Ahmed L, Zakaria S, El Kady N, et al. Schistosomiasis mansoni in an Egyptian village in the Nile Delta. The American journal of tropical medicine and hygiene. 1980;29(5):868-74. Epub 1980/09/01. PubMed PMID: 6969039.

2. Akogun OB, Akogun MK. Human behaviour, water usage and schistosomiasis transmission in a small settlement near Yola, Nigeria. Annals of tropical medicine and parasitology. 1996;90(3):303-11. Epub 1996/06/01. PubMed PMID: 8758144.

3. Arap Siongok TK, Mahmoud AA, Ouma JH, Warren KS, Muller AS, Handa AK, et al. Morbidity in Schistosomiasis mansoni in relation to intensity of infection: study of a community in Machakos, Kenya. The American journal of tropical medicine and hygiene. 1976;25(2):273-84. Epub 1976/03/01. PubMed PMID: 1259088.

4. Babiker SM, Blankespoor HD, Wassila M, Fenwick A, Daffalla AA. Transmission of *Schistosoma haematobium* in North Gezira, Sudan. The Journal of tropical medicine and hygiene. 1985;88(2):65-73. Epub 1985/04/01. PubMed PMID: 4032531.

5. Bartholomew RK, Peters PA, Jordan P. *Schistosoma mansoni* in St. Lucian and Kenyan communities--a comparative study using the Kato stool examination technique. Annals of tropical medicine and parasitology. 1981;75(4):401-5. Epub 1981/08/01. PubMed PMID: 7305508.

6. Birrie H, Abebe F, Gundersen SG, Medhin G, Berhe N, Gemetchu T. Epidemiology of schistosomiasis mansoni in three endemic communities in northeast Ethiopia: Baseline characteristics before endod based intervention. Ethiopian medical journal. 1998;36(2):101-11. PubMed PMID: WOS:000074008200004.

7. Chunge RN, Karumba N, Ouma JH, Thiongo FW, Sturrock RF, Butterworth AE. Polyparasitism in two rural communities with endemic *Schistosoma mansoni* infection in Machakos District, Kenya. The Journal of tropical medicine and hygiene. 1995;98(6):440-4. Epub 1995/12/01. PubMed PMID: 8544228.

8. Coulibaly JT, Ouattara M, Dongo K, Hurlimann E, Bassa FK, Kone N, et al. Epidemiology of intestinal parasite infections in three departments of south-central Cote d'Ivoire before the implementation of a cluster-randomised trial. Parasite epidemiology and control. 2018;3(2):63-76. Epub 2018/05/19. doi: 10.1016/j.parepi.2018.02.003. PubMed PMID: 29774300; PubMed Central PMCID: PMCPMC5952672.

9. Coulibaly JT, N'Gbesso YK, N'Guessan NA, Winkler MS, Utzinger J, N'Goran EK. Epidemiology of schistosomiasis in two high-risk communities of south Cote d'Ivoire with particular emphasis on pre-school-aged children. The American journal of tropical medicine and hygiene. 2013;89(1):32-41. Epub 2013/05/22. doi: 10.4269/ajtmh.12-0346. PubMed PMID: 23690549; PubMed Central PMCID: PMCPMC3748484.

10. Coulibaly JT, N'Gbesso YK, Knopp S, Keiser J, N'Goran EK, Utzinger J. Efficacy and safety of praziquantel in preschool-aged children in an area co-endemic for *Schistosoma mansoni* and *S. haematobium*. PLoS neglected tropical diseases. 2012;6 (12) (no pagination)(e1917).

11. Coulibaly JT, Furst T, Silue KD, Knopp S, Hauri D, Ouattara M, et al. Intestinal parasitic infections in school children in different settings of Cote d'Ivoire: effect of diagnostic approach and implications for control. Parasites & vectors. 2012;5:135. Epub 2012/07/10. doi: 10.1186/1756-3305-5-135. PubMed PMID: 22768986; PubMed Central PMCID: PMCPMC3425256.

12. De Clercq D, Rollinson D, Diarra A, Sacko M, Coulibaly G, Landoure A, et al. Schistosomiasis in Dogon country, Mali: identification and prevalence of the species responsible for infection in the local community. Transactions of the Royal Society of Tropical Medicine and Hygiene. 1994;88(6):653-6. Epub 1994/11/01. PubMed PMID: 7886759.

13. Elmorshedy H, Bergquist R, Abou El-Ela NE, Eassa SM, Elsakka EE, Barakat R. Can human schistosomiasis mansoni control be sustained in high-risk transmission foci in Egypt? Parasites & vectors. 2015;8. doi: 10.1186/s13071-015-0983-2. PubMed PMID: WOS:000357919900001.

14. Erko B, Tedla S, Petros B. Transmission of intestinal schistosomiasis in Bahir Dar, northwest Ethiopia. Ethiopian medical journal. 1991;29(4):199-211. Epub 1991/10/01. PubMed PMID: 1954954.

15. Farag MK, el-Shazly AM, Khashaba MT, Attia RA. Impact of the current National Bilharzia Control Programme on the epidemiology of schistosomiasis mansoni in an Egyptian village. Transactions of the Royal Society of Tropical Medicine and Hygiene. 1993;87(3):250-3. Epub 1993/05/01. PubMed PMID: 8236381.

16. Friis H, Mwaniki D, Omondi B, Muniu E, Magnussen P, Geissler W, et al. Serum retinol concentrations and *Schistosoma mansoni*, intestinal helminths, and malarial parasitemia: a cross-sectional study in Kenyan preschool and primary school children. The American journal of clinical nutrition. 1997;66(3):665-71. Epub 1997/09/01. doi: 10.1093/ajcn/66.3.665. PubMed PMID: 9280190.

17. Garba A, Barkire N, Djibo A, Lamine MS, Sofo B, Gouvras AN, et al. Schistosomiasis in infants and preschool-aged children: Infection in a single *Schistosoma haematobium* and a mixed *S. haematobium-S. mansoni* foci of Niger. Acta tropica. 2010;115(3):212-9.

18. Garba A, Lamine MS, Barkire N, Djibo A, Sofo B, Gouvras AN, et al. Efficacy and safety of two closely spaced doses of praziquantel against *Schistosoma haematobium* and *S. mansoni* and re-infection patterns in school-aged children in Niger. Acta tropica. 2013;128(2):334-44.

19. Gryseels B, Nkulikyinka L. The distribution of *Schistosoma mansoni* in the Rusizi plain (Burundi). Annals of tropical medicine and parasitology. 1988;82(6):581-90. Epub 1988/12/01. PubMed PMID: 3151430.

20. Gryseels B, Polderman AM. The morbidity of schistosomiasis mansoni in Maniema (Zaire). Transactions of the Royal Society of Tropical Medicine and Hygiene. 1987;81(2):202-9. Epub 1987/01/01. PubMed PMID: 3113002.

21. Gundersen SG, Birrie H, Torvik HP, Scherbaum H. Control of *Schistosoma mansoni* in the Blue Nile Valley of western Ethiopia by mass chemotherapy and focal snail control: a primary health care experience. Transactions of the Royal Society of Tropical Medicine and Hygiene. 1990;84(6):819-25. Epub 1990/11/01. PubMed PMID: 2128985.

22. Hiatt RA. Morbidity from *Schistosoma mansoni* infections: an epidemiologic study based on quantitative analysis of egg excretion in two highland Ethiopian villages. The American journal of tropical medicine and hygiene. 1976;25(6):808-17. Epub 1976/11/01. PubMed PMID: 1008126.

23. Hodges MH, Paye J, Koroma MM, Nyorkor ED, Fofonah I, Zhang YB. High level of *Schistosoma mansoni* infection in pre-school children in Sierra Leone highlights the need in targeting this age group for praziquantel treatment. Acta tropica. 2012;124(2):120-5. doi: 10.1016/j.actatropica.2012.07.005. PubMed PMID: WOS:000309372600003.

24. Hodges M, Dada N, Wamsley A, Paye J, Nyorkor E, Sonnie M, et al. Improved mapping strategy to better inform policy on the control of schistosomiasis and soil-transmitted helminthiasis in Sierra Leone. Parasites & vectors. 2011;4(97).

25. Kabatereine NB, Ariho C, Christensen NO. *Schistosoma mansoni* in Pachwach, Nebbi District, Uganda, 40 years after Nelson. Tropical medicine and parasitology : official organ of Deutsche Tropenmedizinische Gesellschaft and of Deutsche Gesellschaft fur Technische Zusammenarbeit (GTZ). 1992;43(3):162-6. Epub 1992/09/01. PubMed PMID: 1470834.

26. Kamel MA, Miller FD, el Masry AG, Zakaria S, Khattab M, Essmat G, et al. The epidemiology of *Schistosoma mansoni*, hepatitis B and hepatitis C infection in Egypt. Annals of tropical medicine and parasitology. 1994;88(5):501-9. Epub 1994/10/01. PubMed PMID: 7979640.

27. Kazura JW, Neill M, Peters PA, Dennis E. Swamp rice farming: possible effects on endemicity of schistosomiasis mansoni and haematobia in a population in Liberia. The American journal of tropical medicine and hygiene. 1985;34(1):107-11. Epub 1985/01/01. PubMed PMID: 3970302.

28. Lakwo TL, Odongo-Aginya EI. The intensity of Schistosoma mansoni in the fishing villages of Bugonga and Kitubulu in Entebbe Peninsula, Uganda. East African medical journal. 1991;68(5):372-7. Epub 1991/05/01. PubMed PMID: 1935732.

29. McCullough FS, Magendantz M. An epidemiological investigation into *Schistosoma mansoni* transmission in Mwanza, Tanzania. Annals of tropical medicine and parasitology. 1974;68(1):69-80. Epub 1974/03/01. PubMed PMID: 4843106.

30. Meurs L, Mbow M, Vereecken K, Menten J, Mboup S, Polman K. Epidemiology of mixed *Schistosoma mansoni* and *Schistosoma haematobium* infections in northern Senegal. International journal for parasitology. 2012;42(3):305-11. Epub 2012/03/01. doi: 10.1016/j.ijpara.2012.02.002. PubMed PMID: 22366733.

31. Nausch N, Dawson EM, Midzi N, Mduluza T, Mutapi F, Doenhoff MJ. Field evaluation of a new antibody-based diagnostic for *Schistosoma haematobium* and *S. mansoni* at the point-of-care in northeast Zimbabwe. BMC infectious diseases. 2014;14:165. Epub 2014/03/29. doi: 10.1186/1471-2334-14-165. PubMed PMID: 24666689; PubMed Central PMCID: PMCPMC4021455.

32. Odogwu SE, Ramamurthy NK, Kabatereine NB, Kazibwe F, Tukahebwa E, Webster JP, et al. *Schistosoma mansoni* in infants (aged < 3 years) along the Ugandan shoreline of Lake Victoria. Annals of tropical medicine and parasitology. 2006;100(4):315-26. doi: 10.1179/136485906x105552. PubMed PMID: 16762112.

33. Stothard JR, Kabatereine NB, Tukahebwa EM, Kazibwe F, Mathieson W, Webster JP, et al. Field evaluation of the Meade Readiview handheld microscope for diagnosis of intestinal schistosomiasis in Ugandan school children. The American journal of tropical medicine and hygiene. 2005;73(5):949-55. Epub 2005/11/12. PubMed PMID: 16282310.

34. Ouma JH, Wijers DJ, Arap Siongok TK. The effect of repeated targeted mass treatment on the prevalence of schistosomiasis mansoni and the intensity of infection in Machakos, Kenya. Annals of tropical medicine and parasitology. 1985;79(4):431-8. Epub 1985/08/01. PubMed PMID: 3935065.

35. Polderman AM, Mpamila K, Manshande JP, Bouwhuis-Hoogerwerf ML. Methodology and interpretation of parasitological surveillance of intestinal schistosomiasis in Maniema, Kivu Province, Zaire. Annales de la Societe belge de medecine tropicale. 1985;65(3):243-9. Epub 1985/09/01. PubMed PMID: 3935059.

36. Raso G, Luginbuhl A, Adjoua CA, Tian-Bi NT, Silue KD, Matthys B, et al. Multiple parasite infections and their relationship to self-reported morbidity in a community of rural Cote d'Ivoire. International Journal of Epidemiology. 2004;33(5):1092-102.

37. Stelma FF, Talla I, Polman K, Niang M, Sturrock RF, Deelder AM, et al. Epidemiology of *Schistosoma mansoni* infection in a recently exposed community in northern Senegal. The American journal of tropical medicine and hygiene. 1993;49(6):701-6. Epub 1993/12/01. PubMed PMID: 8279638.

38. Verani JR, Abudho B, Montgomery SP, Mwinzi PNM, Shane HL, Butler SE, et al. Schistosomiasis among young children in Usoma, Kenya. American Journal of Tropical Medicine and Hygiene. 2011;84(5):787-91.

39. Wiselka MJ, Robinson MB, Clipsham K, Weddon S. The epidemiology of schistosomiasis in central Malawi. East African medical journal. 1988;65(2):102-7. Epub 1988/02/01. PubMed PMID: 3135991.

40. Zaki A, Bassili A, Amin G, Aref T, Kandil M, Abou Basha LM. Morbidity of schistosomiasis mansoni in rural Alexandria, Egypt. Journal of the Egyptian Society of Parasitology. 2003;33(3):695-710. Epub 2004/01/08. PubMed PMID: 14708847.

41. Amuta EU, Houmsou RS. Prevalence, intensity of infection and risk factors of urinary schistosomiasis in pre-school and school aged children in Guma Local Government Area, Nigeria. Asian Pacific journal of tropical medicine. 2014;7(1):34-9. Epub 2014/01/15. doi: 10.1016/s1995-7645(13)60188-1. PubMed PMID: 24418080.

42. Anosike JC, Oguwuike U, Nwoke B, Asor J, Ikpeama C, Nwosu D, et al. Studies on vesical schistosomiasis among rural Ezza farmers in the southwestern border of Ebonyi State, Nigeria. Annals of agricultural and environmental medicine : AAEM. 2006;13(1):13-9. Epub 2006/07/18. PubMed PMID: 16841866.

43. Anosike JC, Okere AN, Nwoke BEB, Chukwu JU, Nwosu DC, Njoku-Tony RF, et al. Endemicity of vesical schistosomiasis in the Ebonyi Benue River valley, South Eastern Nigeria. International journal of hygiene and environmental health. 2003;206(3):205-10. doi: 10.1078/1438-4639-00214.

44. Ben SA, Useh MF. A comparative study on the efficacy of praziquantel and albendazole in the treatment of urinary schistosomiasis in Adim, Cross River State, Nigeria. International health. 2017;9(5):288-93. Epub 2017/09/16. doi: 10.1093/inthealth/ihx031. PubMed PMID: 28911129.

45. Campbell SJ, Stothard JR, O'Halloran F, Sankey D, Durant T, Ombede DE, et al. Urogenital schistosomiasis and soiltransmitted helminthiasis (STH) in Cameroon: An epidemiological update at Barombi Mbo and Barombi Kotto crater lakes assessing prospects for intensified control interventions. Infectious diseases of poverty. 2017;6. doi: 10.1186/s40249-017-0264-8. PubMed PMID: WOS:000397737700001.

46. Clark TE, Appleton CC, Kvalsvig JD. Schistosomiasis and the use of indigenous plant molluscicides: a rural South African perspective. Acta tropica. 1997;66(2):93-107. Epub 1997/08/01. PubMed PMID: 9227802.

47. Deribe K, Eldaw A, Hadziabduli S, Kailie E, Omer MD, Mohammed AE, et al. High prevalence of urinary schistosomiasis in two communities in South Darfur: implication for interventions. Parasites & vectors. 2011;4:14. Epub 2011/02/09. doi: 10.1186/1756-3305-4-14. PubMed PMID: 21299881; PubMed Central PMCID: PMCPMC3042003.

48. Duke BO, Moore PJ. The use of a molluscicide in conjunction with chemotherapy to control *Schistosoma haematobium* at the Barombi Lake foci in Cameroon. II. Urinary examination methods, the use of niridazole to attack the parasite in man, and the effect on transmission from man to snail. Tropenmedizin und Parasitologie. 1976;27(4):489-504. Epub 1976/12/01. PubMed PMID: 1006804.

49. Ejezie GC, Uko IE, Braide EI. Schistosomiasis in Cross River State, Nigeria: 1. Prevalence and intensity of infection in Adim, Akamkpa Local Government Area. Journal of hygiene, epidemiology, microbiology, and immunology. 1991;35(2):141-7. Epub 1991/01/01. PubMed PMID: 1940328.

50. Garba A, Pion S, Cournil A, Milet J, Schneider D, Campagne G, et al. Risk factors for *Schistosoma haematobium* infection and morbidity in two villages with different transmission patterns in Niger. Acta tropica. 2010;115(1-2):84-9.

51. Goatly KD, Jordan P. Schistosomiasis in Zanzibar and Pemba. East African medical journal. 1965;42:1-9. Epub 1965/01/01. PubMed PMID: 14271180.

52. Houmsou RS, Agere H, Wama BE, Bingbeng JB, Amuta EU, Kela SL. Urinary Schistosomiasis among Children in Murbai and Surbai Communities of Ardo-Kola Local Government Area, Taraba State, Nigeria. Journal of tropical medicine. 2016;2016:9831265. Epub 2017/01/18. doi: 10.1155/2016/9831265. PubMed PMID: 28096819; PubMed Central PMCID: PMCPMC5206853.

53. King CL, Miller FD, Hussein M, Barkat R, Monto AS. Prevalence and intensity of *Schistosoma haematobium* infection in six villages of upper Egypt. The American journal of tropical medicine and hygiene. 1982;31(2):320-7.

54. Klumpp RK, Webbe G. Focal, seasonal and behavioural patterns of infection and transmission of *Schistosoma haematobium* in a farming village at the Volta Lake, Ghana. The Journal of tropical medicine and hygiene. 1987;90(5):265-81. Epub 1987/10/01. PubMed PMID: 3118054.

55. Koura M, Upatham ES, Awad AH, Ahmed MD. Prevalence of *Schistosoma haematobium* in the Koryole and Merca Districts of the Somali Democratic Republic. Annals of tropical medicine and parasitology. 1981;75(1):53-61.

56. Lyons GR. Schistosomiasis in north-western Ghana. Bulletin of the World Health Organization. 1974;51(6):621-32. Epub 1974/01/01. PubMed PMID: 4549612; PubMed Central PMCID: PMCPMC2366268.

57. Mansour NS, Higashi GI, Schinski VD, Murrell KD. A longitudinal study of *Schistosoma haematobium* infection in Qena governorate, Upper Egypt. 1. Initial epidemiological findings. The American journal of tropical medicine and hygiene. 1981;30(4):795-805. Epub 1981/07/01. PubMed PMID: 7258491.

58. Mekonnen A, Legesse M, Belay M, Tadesse K, Torben W, Teklemariam Z, et al. Efficacy of Praziquantel against *Schistosoma haematobium* in Dulshatalo village, western Ethiopia. BMC research notes. 2013;6:392. Epub 2013/10/01. doi: 10.1186/1756-0500-6-392. PubMed PMID: 24073761; PubMed Central PMCID: PMCPMC3849756.

59. Midzi N, Butterworth AE, Mduluza T, Munyati S, Deelder AM, van Dam GJ. Use of circulating cathodic antigen strips for the diagnosis of urinary schistosomiasis. Transactions of the Royal Society of Tropical Medicine and Hygiene. 2009;103(1):45-51. Epub 2008/10/28. doi: 10.1016/j.trstmh.2008.08.018. PubMed PMID: 18951599.

60. Morenikeji O, Quazim J, Omoregie C, Hassan A, Nwuba R, Anumudu C, et al. A cross-sectional study on urogenital schistosomiasis in children; haematuria and proteinuria as diagnostic indicators in an endemic rural area of Nigeria. African health sciences. 2014;14(2):390-6. Epub 2014/10/17. doi: 10.4314/ahs.v14i2.15. PubMed PMID: 25320589; PubMed Central PMCID: PMCPMC4196391.

61. Mott KE, Dixon H, Osei-Tutu E, England EC, Ekue K, Tekle A. Indirect screening for *Schistosoma haematobium* infection: a comparative study in Ghana and Zambia. Bulletin of the World Health Organization. 1985;63(1):135-42. Epub 1985/01/01. PubMed PMID: 3872732; PubMed Central PMCID: PMCPMC2536345.

62. Wami WM, Nausch N, Midzi N, Gwisai R, Mduluza T, Woolhouse M, et al. Identifying and evaluating field indicators of urogenital schistosomiasis-related morbidity in preschool-aged children. PLoS neglected tropical diseases. 2015;9(3):e0003649. Epub 2015/03/21. doi: 10.1371/journal.pntd.0003649. PubMed PMID: 25793584; PubMed Central PMCID: PMCPMC4368198.

63. Mutapi F, Rujeni N, Bourke C, Mitchell K, Appleby L, Nausch N, et al. *Schistosoma haematobium* treatment in 1-5 year old children: safety and efficacy of the antihelminthic drug praziquantel. PLoS neglected tropical diseases. 2011;5(5):e1143. PubMed PMID: 21610855.

64. Schutte CH, Van Deventer JM, Eriksson IM. Parasitic infections in Black children in an endemic schistosomiasis area in Natal. South African medical journal = Suid-Afrikaanse tydskrif vir geneeskunde. 1977;51(9):268-72. Epub 1977/02/26. PubMed PMID: 191941.

65. Sousa-Figueiredo JC, Basanez MG, Mgeni AF, Khamis IS, Rollinson D, Stothard JR. A parasitological survey, in rural Zanzibar, of pre-school children and their mothers for urinary schistosomiasis, soil-transmitted helminthiases and malaria, with observations on the prevalence of anaemia. Annals of tropical medicine and parasitology. 2008;102(8):679-92. Epub 2008/11/13. doi: 10.1179/136485908x337607. PubMed PMID: 19000385.

66. Rudge J, Stothard R, Basanez M, Mgeni A, Khamis S, Khamis A, et al. Micro-epidemiology of urinary schistosomiasis in Zanzibar: Local risk factors associated with distribution of infections among schoolchildren and relevance for control. Acta tropica. 2008;105(1):45 - 54. doi: <https://doi.org/10.1016/j.actatropica.2007.09.006>.

67. Verle P, Stelma F, Desreumaux P, Dieng A, Diaw O, Kongs A, et al. Preliminary study of urinary schistosomiasis in a village in the delta of the Senegal river basin, Senegal. Transactions of the Royal Society of Tropical Medicine and Hygiene. 1994;88(4):401-5. Epub 1994/07/01. PubMed PMID: 7570817.

68. Wilkins HA, Goll PH, Moore PJ. *Schistosoma haematobium* infection and haemoglobin concentrations in a Gambian community. Annals of tropical medicine and parasitology. 1985;79(2):159-61. Epub 1985/04/01. PubMed PMID: 3938202.
